# Resetting and Entrainment of Reentrant Arrhythmias: Part I: Concepts, Recognition, and Protocol for Evaluation: Surface ECG versus Intracardiac Recordings

**DOI:** 10.1111/pace.12064

**Published:** 2013-01-10

**Authors:** JESÚS ALMENDRAL, RAÚL CAULIER-CISTERNA, JOSÉ LUIS ROJO-ÁLVAREZ

**Affiliations:** * Cardiac Arrhythmia UnitGrupo Hospital de Madrid, Universidad CEU-San PabloMadrid, Spain; † Department of Signal Theory and Communications, University Rey Juan CarlosFuenlabrada, Spain

**Keywords:** Resetting, entrainment, reentry, tachycardia, fusion, pacing

## Abstract

In this paper, we review the information accumulated over the years regarding the phenomena of resetting and entrainment of reentrant arrhythmias. Over three decades of research and clinical applications, these phenomena have demonstrated that they stay as a main tool for an intellectual understanding of reentry and to base strategies for localization of critical areas for ablative therapies. This review will be divided into two parts. This first part deals with the bases for the concept development, the means for the detection of these phenomena, and their mechanistic implications. Resetting is described as a particular response of a given rhythm to an external perturbation, indicating interaction between them. Entrainment indicates continuous reset of the rhythm when the perturbation is repetitive. The mechanisms that explain these responses in reentrant rhythms are presented. Fusion, both at the surface electrocardiogram and at the level of intracardiac recordings, is discussed in detail, with its value and limitations as a key concept to recognize entrainment and reentry. Computer simulations are used as an aid to a better understanding. Differences between resetting and entrainment are considered, and a pacing protocol to study these phenomena described.

## The Concept of Resetting and the Tachycardia Clock

**Figure 1:**
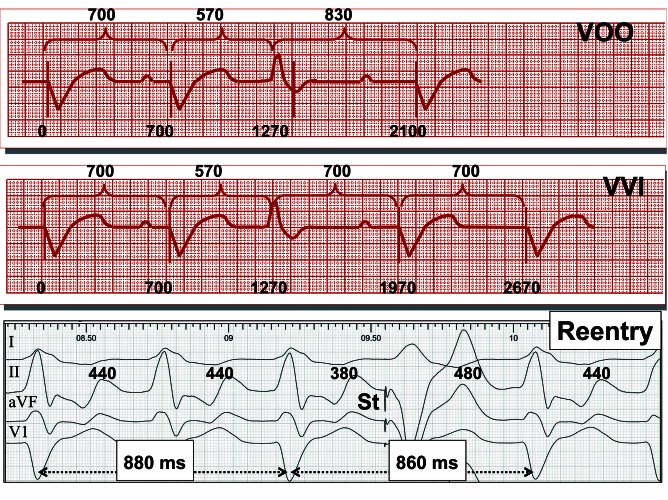
Illustrative tracings of resetting of a pacemaker rhythm and of a reentrant arrhythmia. Upper and mid panels: a premature ventricular contraction occurs during a pacemaker rhythm: if it is not sensed (VOO mode) the rhythm will not be reset; in contrast, if the premature ventricular contraction is sensed (VVI mode), it will interact with the pacemaker, and the rhythm will be reset. Bottom panel: a single ventricular extrastimulus (St) is delivered during a left free wall orthodromic accessory pathway-mediated tachycardia (with left bundle branch block). The tachycardia is reset because the sum of the two intervals encompassing the extrastimulus (860 ms) is less than twice the tachycardia cycle length (880 ms). So the 480-ms pause after the extrastimulus is less than fully compensatory.

The term resetting comes from engineering and is used when “a system (a clock for example) is restored back to zero.” Resetting of a cardiac rhythm can be easily understood if one compares what is seen in the electrocardiogram (ECG) of a patient with a ventricular pacemaker after a ventricular premature contraction if the pacemaker is programmed in the VOO mode or in the VVI mode (Fig. 1, upper and mid panels). In both cases, the pacemaker rhythm is resumed after the ventricular premature contraction. Since the ventricular premature contraction is not sensed in the VOO mode the pause after the ventricular premature contraction has to be fully compensatory so “the clock of the rhythm” is ultimately unaltered. In contrast, in the VVI mode, the ventricular premature contraction is sensed by the pacemaker so the next pacing stimulus is advanced. The relative pause that follows is less than fully compensatory. The same can occur in reentrant rhythms (Fig. 1, bottom panel). The consequence is that “the clock of the rhythm” has changed permanently despite the rhythm being identical, the rhythm has been reset.

## The Essence of Resetting: Interaction between the Cardiac Rhythm and the Perturbation Generated

In the example of Figure 1, the reason why the ventricular premature contraction produces a noncompensatory pause in the mid panel is because there is an interaction between the activation wavefront generated by the ventricular premature contraction and the electronic pacemaker circuit that senses this activation wavefront as it reaches the site where the electrode is located and generates the next pacemaker stimulus accordingly to the pacemaker discharge cycle. Such an interaction is not possible in the VOO mode.

**Figure 2:**
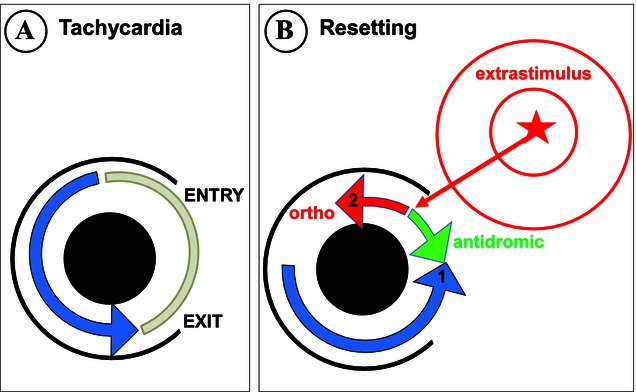
Schematic diagram illustrating the mechanism of resetting in reentrant arrhythmias. Panel A represents a circular reentrant circuit. Most of it is surrounded by a black circumferencial barrier, that is, interrupted on its right side. The black area inside the circuit represents unexcitable tissue. The white area around the circuit would be the remaining of the heart (the cardiac chamber). Inside the circuit electrical activation is taking place, represented by the blue arrowhead, that is followed by a tail of refractoriness (blue area). So all the blue area is not excitable at this precise moment. The tissue in the circuit that is excitable, usually referred to as excitable gap, is depicted in light gray. As the activation inside the circuit proceeds, it is expected that it will be confined in the circuit only as long as a barrier exists, so it will exit to the surrounding myocardium as soon as there is no barrier (exit site, “exit” in the figure). If activation wavefronts generated outside the circuit approach it, they would activate the tissue inside the circuit if there is no barrier and if the tissue is excitable. So the entry site would be the closest site to the external activation wavefront that is not surrounded by barrier and that is excitable. Considering the situation in panel B, where the wavefront is generated geometrically closer to the upper boundary of the barrier, the entry site would be its most superior end, as represented in panel A (“entry”). Panel B represents the situation several milliseconds later. An extrastimulus has been delivered at a site away from the circuit. The corresponding wavefront has reached the reentrant circuit at a time the tissue was excitable (small red arrow). Activation inside the circuit proceeds both in the direction of activation during tachycardia (orthodromic wavefront, “ortho” in the figure) and in the opposite direction (antidromic wavefront, “antidromic” in the figure). Since the antidromic wavefront collides with the activation wavefront inside the circuit, the final result is that the activation jumps from site 1 to site 2, thus shortcircuiting the circuit (see text for further explanations).

In the case of reentrant circuits the phenomena underlining resetting are more complex, as schematically presented in Figure 2. If an extrastimulus is delivered at an adequate site and with an adequate timing, the resultant wavefront (Fig. 2, panel B) may access and penetrate the circuit. As a result, two wavefronts are generated usually called orthodromic and antidromic, according to whether they proceed in the same or the opposite direction as during tachycardia. As can be appreciated in Figure 2, the antidromic wavefront is necessarily destined to collide with the activation wavefront from the tachycardia (blue arrowhead), and both wavefronts will extinguish. So the only wavefront that will keep proceeding is the orthodromic wavefront. The final result, as depicted in Figure 2, is that when the activation inside the circuit is at site 1, in reality it is at site 2, so the activation has “jumped” over a certain distance (distance between site 1 and site 2) inside the circuit, so it will take less time to arrive to the exit site of the circuit next time, so the activation will be advanced (reset, the tachycardia clock will be advanced) for just one beat. Since the circuit has not changed, if no more external perturbations occur, the following beats will appear with intervals identical to the tachycardia cycle length (TCL) but having now the reset beat as the time reference.

From the above discussion, it becomes clear that for resetting to occur there has to be an interaction between the basic rhythm (tachycardia) and an external perturbation (either artificially generated, such as programmed extrastimuli, or naturally occurring such as a premature atrial or ventricular contraction), and that resetting results from a peculiar type of interaction: change in the tachycardia clock but maintenance of the tachycardia circuit. Other examples of interaction, as presented in Table I, include termination of the reentrant rhythm (tachycardia termination), or a change in the basic mechanism (different tachycardia).

**Table I:** Types of Response to Pacing Maneuvers during Reentrant Arrhythmias

Response Type	Pacing Type	Interaction with Reentry	Recognition
Resetting	Extrastimuli (Single or double)	Yes	Pause different from compensatory
Entrainment	Continuous pacing	Yes, continuous	Constant fusion, recordings from the circuit
Tachycardia termination	Extrastimuli, continuous pacing	Yes, intermittent	Tachycardia termination
Concealed perpetuation	Extrastimuli, continuous pacing	No	Compensatory pause, variable fusion
Change in tachycardia	Extrastimuli, continuous pacing	Yes, intermittent	Different tachycardia after pacing
Stopping and re-starting tachycardia	Continuous pacing	Yes, intermittent	Response depends on number of paced impulses
Overdrive suppression	Continuous pacing	Does not occur in reentry	Long variable pause, no fusion
Overdrive acceleration	Continuous pacing	Rare in reentry	The faster the pacing the more it accelerates

## The Essence of Transient Entrainment: Continuous Resetting, Continuous Interaction: All the Tissue at the Paced and Circuit Chamber is Accelerated to the Pacing Rate

Let us imagine that in the situation represented in Figure 2 (panel B), after the orthodromic wavefront has traveled a good way along the reentrant circuit, for example when it is reaching the exit site, a second paced stimulus is delivered at the pacing site; similar phenomena will take place and the previously reset circuit will be reset again. If pacing continues at the pacing site at a constant rate, a little faster than the tachycardia rate, each pacing stimulus could arrive at the entry site of the circuit a little earlier than the activation wavefront of the tachycardia itself (in reality the orthodromic wavefront of the previous paced beat). This could be a perfectly stable situation as long as the pacing rate is constant and each orthodromic wavefront propagates all the way along the reentrant circuit. In this situation, if the chamber where the circuit is located is observed, that is, with an ECG and/or multiple intracardiac recordings, the activation rate at all sites equals the pacing rate, but the tachycardia configuration subsists in a certain way, so if pacing is stopped at any time, the tachycardia will resume unaltered, at its original rate; this was originally described by Waldo et al. with the expression: “the tachycardia has been transiently entrained at the pacing rate.”^1^,^2^ Josephson described entrainment as a repetitive resetting of the previously reset circuit.^3^ The reason why the tachycardia will continue when pacing is stopped is because there will be no paced wavefront, so no antidromic wavefront with which to collide; thus, the tachycardia wavefront will proceed uninterrupted all the way through the reentrant circuit at its original rate.

**Figure 3:**
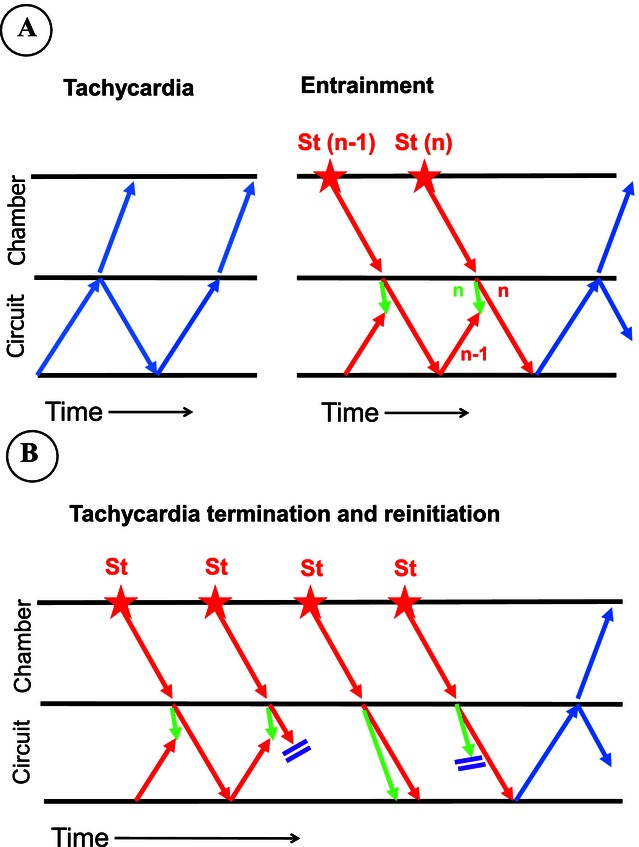
Activation during tachycardia and during pacing is schematically depicted in a ladder diagram format with time as the X-axis. The tissue is schematically divided into the reentrant circuit (“circuit”) and the remaining of the chamber (“chamber”). Panel A represents activation during tachycardia (left) and during the last two beats (n – 1 and n beats) of a pacing train, introduced at a distance from the chamber, producing entrainment (right). Blue arrows represent activation during tachycardia in an ondulating format to represent circular continuous activation. During entrainment, each paced wavefront (St) propagates through the intervening tissue (red arrow in chamber). As it enters the circuit, it generates an orthodromic wavefront (red arrows in the circuit) and an antidromic wavefront (green arrows in the circuit); the latter collides with the activation resulting from the orthodromic wavefront of the previous paced beat (“n green” collides with “n – 1 red”). Please note that, during entrainment, all the tissue within the reentrant circuit is accelerated at the pacing cycle length despite conduction velocity being the same as during tachycardia. See text for further discussion. Panel B represents pacing-induced tachycardia termination and reinitiation. Tachycardia terminates in the second beat due to block of the orthodromic wavefront inside the circuit. The fourth paced impulse blocks in the antidromic direction so tachycardia reinitiates. If pacing would have been stopped after the second or third paced impulse, the tachycardia would have been terminated, but as it is stopped after the fourth beat, the tachycardia continues. Please note that despite activation of the intervening tissue (“chamber”) at the pacing rate, some areas of the reentrant circuit are not activated at that rate.

**Figure 4:**
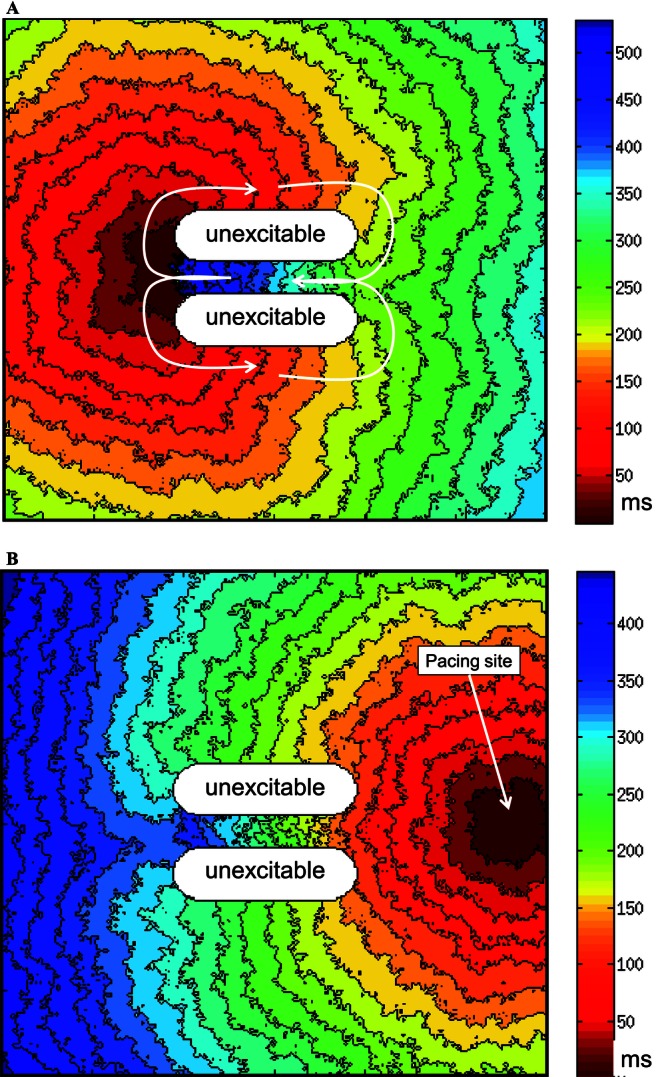
Computer simulation of a 2-dimensional figure-of-eight reentrant model to study resetting and entrainment. Panel A: electrical activation during reentry as taken from Video 1 (cycle length is approximately 550 ms). Activation takes place around two anatomical obstacles of unexcitable tissue, and is represented as isochrones in a color-coded fashion. White arrows represent “the circuit,” which is protected in the small area comprised by the two unexcitable barriers. Panel B: The same tissue in the absence of reentry is now activated by a paced wavefront generated at its right margin at a paced cycle length of 450 ms.

**Figure 5:**
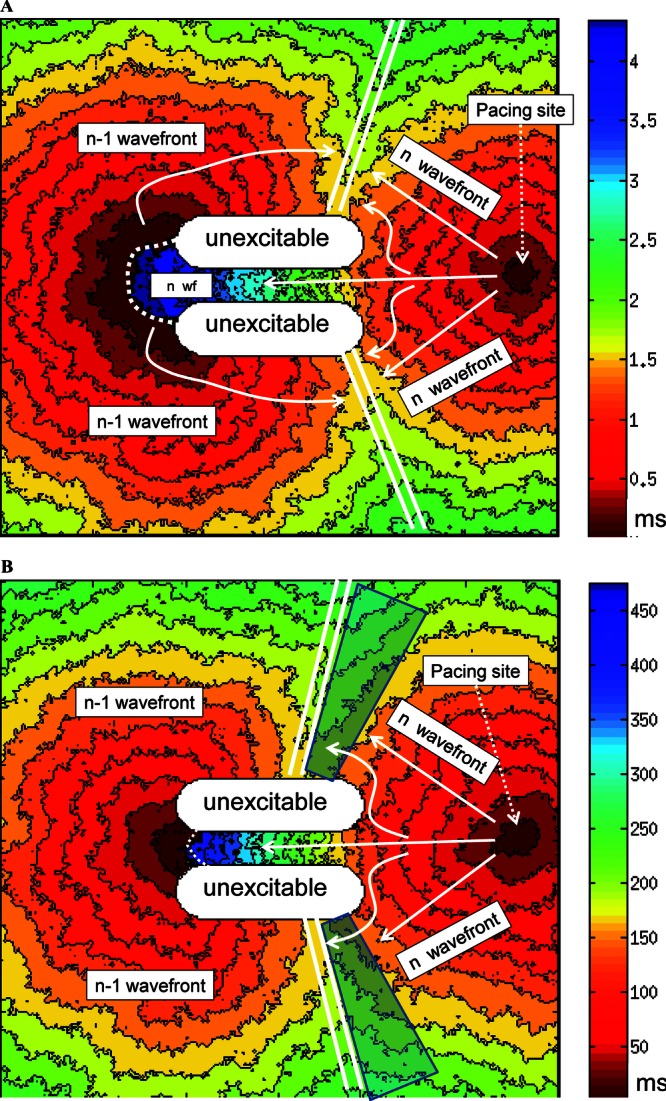
Computer simulation of activation during a paced beat entraining the reentrant rhythm, as seen in Video 1. The format is similar to that in Figure 4 A. The difference between panels A and B is that the paced cycle length is 450 ms in panel A and 350 ms in panel B. In both panels the reference time is taken when the pacing stimulus occurs (red), but at this same time the impulse from the n – 1 wavefront have exited from the protected part of the circuit (also red at n – 1 wavefront). Comparing panel A with Figure 4 B, that depicts activation from the same site and rate, it seems obvious that activation is different because of fusion. Collision of both wavefronts is depicted by the parallel white lines in both panels. The white curved small arrows represent antidromic penetration at the nonprotected part of the circuit. Note that the degree of fusion is related to the pacing rate, a larger part of the preparation (both inside and outside the circuit) is captured by the “n” paced wavefront (antidromically) in panel B. The translucent areas in panel B represent the only areas that are captured orthodromically at the slower rate but antidromically at the faster rate, and thus will be the areas where the fourth criterion could be met (see text for discussion).

What is unique to this situation is that each paced beat interacts with the tachycardia wavefront and resets the circuit. By virtue of this interaction, all the tissue of the chamber where the circuit is located (including the tissue of the reentrant circuit itself) is activated at the pacing rate, either by the antidromic wavefront of the paced beat, the orthodromic wavefront of the paced beat, or by the orthodromic wavefront of the preceding beat (after having proceeded along the tachycardia circuit). This is schematically shown in Figure 3A, that depicts activation in a ladder diagram format to include time as the X-axis. But also unique to this situation is that the tachycardia somehow remains “alive” so that if pacing is stopped at any moment the tachycardia will continue unaltered. This differentiates transient entrainment from stopping and re-starting the tachycardia by pacing (Fig. 3B), another form of interaction between pacing and tachycardia (Table I). In the latter, after pacing is interrupted, the tachycardia may continue, but only if pacing is stopped at the adequate time, otherwise the tachycardia will not appear. Moreover, as shown in Figure 3B, not all the tissue of the circuit is continuously activated at the pacing rate. However, the differentiation between transient entrainment and stopping and re-starting tachycardia may not be easy (see later). This triad, beat-to-beat interaction between the paced and tachycardia wavefronts, activation of all the tissue in the chamber where the circuit is located at the pacing rate, and “persistence” of the tachycardia during pacing, represents the three essential components of the phenomenon of entrainment of reentrant arrhythmias. In the case of resetting, since only one beat interacts with the circuit, it will not be activated at the pacing rate; in this situation, an advancement of the activation will represent the same finding. To provide a better understanding, both spatially and temporally, we have developed a 2-dimensional computer simulation of a figure-of-eight reentrant pathway around two unexcitable obstacles (Fig. 4A), during which pacing produced resetting and entrainment, as can be observed in Video 1. Figure 5A represents the activation map during one of the paced beats that entrained the tachycardia in Video 1.

It is interesting to note that, despite all the tissue in the chamber undergoing a higher number of electrical activations per minute (is accelerated), conduction velocity does not increase in any part of the chamber (it could even decrease). This apparent paradox, particularly in relation to the reentrant circuit, is explained because part of the tissue in the reentrant circuit is occupied in a different way as during tachycardia, as depicted in Figure 2; the area of the reentrant circuit occupied by the antidromic wavefront occupies the circuit in a different way (a different direction) but at the same time as other parts of the circuit are being activated (by the orthodromic wavefront, in fact by two orthodromic wavefronts, that are generated by the present paced beat [n beat] and by the previous paced beat [n – 1 beat] after it has proceeded along the reentrant circuit, Figure 3A). The fact that several parts of the reentrant circuit are activated at the same time by different wavefronts explains the apparent paradox of acceleration in depolarization rate but not in conduction velocity.

## Recognition of Resetting and Entrainment: Electrocardiographic Fusion for Recognition of Entrainment

Resetting is easily recognized because it is easy to detect whether the pause after one or two extrastimuli is fully compensatory (usually less than but occasionally more than fully compensatory). In contrast, after continuous pacing with a significant number of paced beats in a row, it is difficult if not impossible to find out if the postpacing interval (PPI) is compensatory or not.

The essence of the phenomenon, as stated above, could be demonstrated by extensive mapping of the paced chamber, with recordings from inside the circuit, but even after that a definite conclusion may be difficult to reach and overall, this is frequently impractical in clinical electrophysiology.

**Figure 6:**
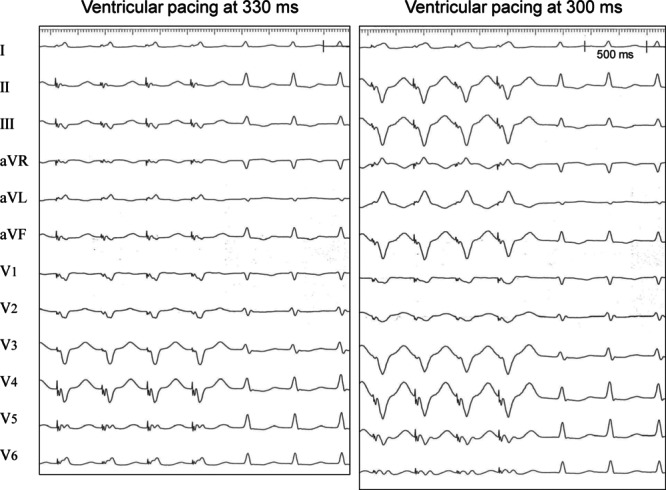
Example of constant and progressive fusion. Both panels illustrate entrainment of an accessory pathway-mediated tachycardia by right apical ventricular pacing at two constant pacing rates (330 ms on the left and 300 ms on the right panel). In both panels the first four QRS complexes are paced and show a constant morphology. In the left panel the paced QRS morphology is totally unexpected for an apical paced site, and the QRS is narrower than in the right panel, because the paced QRS are fused (constant fusion). Please note a dramatic difference in the morphology of the paced QRS complex in relation to pacing rate (progressive fusion).

**Figure 7:**
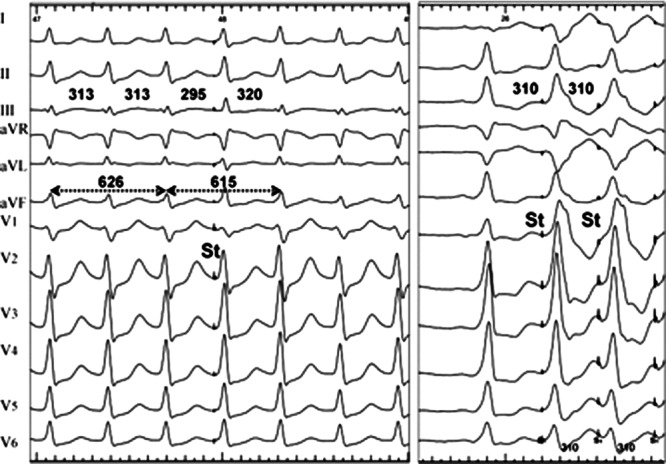
Resetting with fusion. Left panel: a left ventricular extrastimulus (St) is delivered during a regular orthodromic tachycardia mediated by a left-sided accessory pathway. Right panel: left ventricular stimulation at identical site and rate is started during sinus rhythm. Note that the extrastimulus resets the tachycardia, but the QRS is totally different from stimulation during sinus rhythm at the same site (right panel); the QRS of the extrastimulus is a fused QRS complex, actually resembling more the tachycardia QRS morphology (there are only minor changes—see III, aVL, and aVF) and duration (is narrow). Measurements were obtained at high screen speed for accuracy. Courtesy of Dr. M. Pachon and Dr. M.A. Arias.

**Figure 8:**
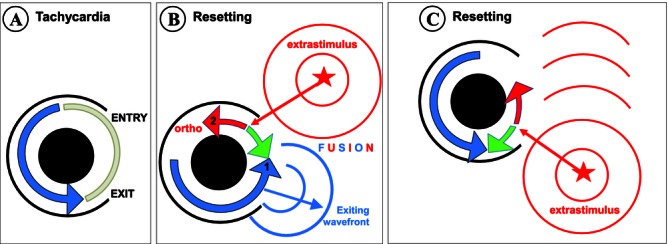
Schematic representation of resetting/entrainment with (and without) electrocardiographic fusion. The format is similar to that of Figure 2, but now activation by the tachycardia exiting wavefront is represented. Note that global activation of the tissue outside the circuit (area to the right of the circuit in panel B) results from fusion of two activation wavefronts. Panel C illustrates that, pacing from a different site, the tachycardia will be entrained, but no fusion outside the circuit will occur. See text for discussion.

**Figure 9:**
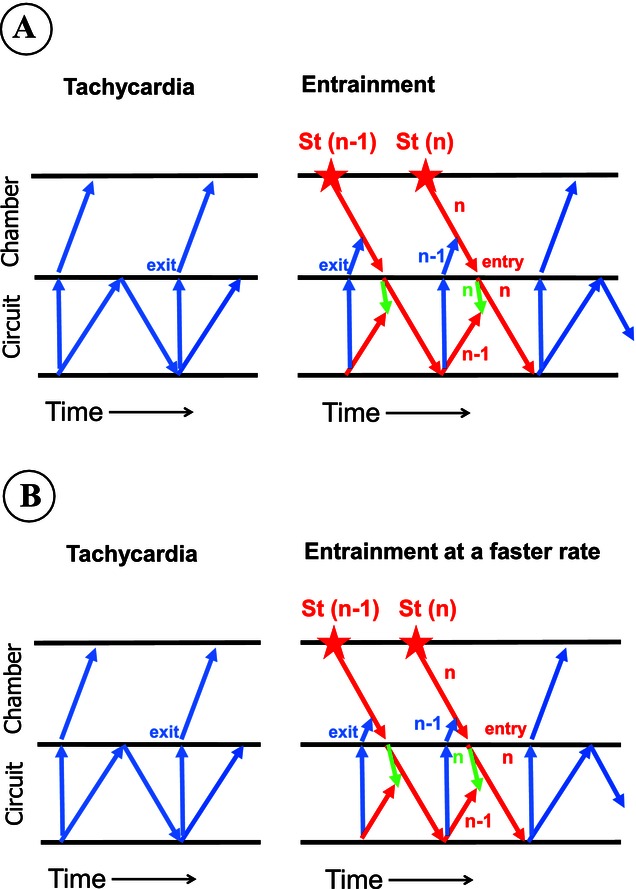
Schematic representation of entrainment with electrocardiographic fusion in a ladder diagram. The format in both panels is similar to that of Figure 3A, but the exit from the tachycardia is now depicted as a “tunnel-like” structure from the circuit, to allow the representation of fusion in a 1-dimensional scheme (in the right panel). Note that fusion in the activation of the chamber occurs because activation resultant from the “n – 1” paced wavefront, as it exits orthodromically from the circuit, is coincidental in time with the “n” paced wavefront. The comparison of panel A with panel B illustrates progressive fusion. As the pacing rate increases (panel B), since each paced wavefront is generated sooner after the previous impulse, more myocardial mass of the chamber will be activated by the paced wavefront and less due to the exiting wavefront from the tachycardia, so the degree of fusion will change, and this will be reflected in the electrocardiogram. Please also note that the antidromic wavefront in the circuit will also invade a greater proportion of the tissue in the circuit.

Waldo et al. made the seminal observation that continuous pacing during reentrant tachycardia followed by persistence of the tachycardia was sometimes associated with the phenomenon of constant fusion as recognized in the surface ECG.^4^,^5^ They proposed that this observation could demonstrate entrainment and was formulated as two criteria for the recognition of entrainment (they also proposed two additional entrainment criteria, see later): (1) “when pacing at a constant rate that is faster than the rate of the tachycardia and which fails to interrupt it, there is the demonstration of constant fusion beats in the ECG except for the last captured beat, which is not fused”; (2) “during a tachycardia, when pacing at two or more constant rates that are faster than the rate of the tachycardia but which fails to interrupt it, there is the demonstration of constant fusion beats in the ECG at each rate, but different degrees of constant fusion at each rate”^4^,^5^ (Fig. 6). This last criterion is usually referred to as progressive fusion. Our group described that fusion can also occur in association with resetting having similar implications^6^ (Fig. 7). The explanation for these criteria is depicted in Figures 5A and 8: if the orthodromic wavefront inside the circuit in Figure 2 accessed the exit site of the circuit, it is expected that it exits to the surrounding myocardium as demonstrated in Figure 8, and this will occur at the same time as the paced wavefront approaches the circuit, so the activation of the myocardium in the chamber where the circuit is located, but out of it, will be the result of two different wavefronts (Figs. 5A and 8), that is, a fused beat. Figure 9A depicts these same phenomena in a complementary fashion, using the ladder diagram that provides an idea of time. Please note that the exiting wavefront from the circuit results from the activation that has traveled through the circuit, but had ultimately originated from the previous paced beat. So, after the last paced beat, the first tachycardia beat depends on that last paced wavefront, in that sense it is entrained, and obviously not fused because there is no more pacing. However, some confusion was created when it was suggested that this last entrained beat occurs “at the pacing rate.”^7^ As later demonstrated in human VT, this only occurs in less than 5% of VT.^8^

Why is constant fusion in the surface ECG so important? Because it certifies the 1:1 interaction between the paced wavefront and the circuit and because it certifies that the tachycardia “is still alive,” two critical components of the essence of the phenomenon of entrainment as stated before. If each orthodromic wavefront not only enters the circuit and proceeds through it, but exits from it (activating part of the chamber and producing fusion), this means both that the interaction has taken place and that the tachycardia is alive. In addition, if the whole circuit and the intervening tissue have been activated at the pacing cycle length, this is evidence that the whole chamber is activated at the pacing rate.

How to detect fusion? A deeper discussion on fusion follows (see later) but let us mention by now that a mere change in the QRS/P-wave contour during pacing is not enough. Pacing will always change the QRS/P-wave morphology as long as it captures the myocardium and the pacing site is located away from the circuit. The way to detect fusion is by comparing the QRS/P-wave contour during pacing when the tachycardia is present with the QRS/P-wave contour during pacing at identical site and rate but in the absence of tachycardia (compare Figs. 4B, 5A, and 7). Since this is not always easy to obtain, the second criterion formulated by Waldo et al.,^4^,^5^ is useful because if progressive fusion can be demonstrated this obviates the need for stimulation in the absence of tachycardia. Figures 5 and 9 illustrate the mechanism of progressive fusion: as the pacing rate increases, the paced wavefront is expected to activate a greater proportion of the chamber mass and the exiting wavefront from the circuit a lower proportion of it, so the degree of fusion will change in the electrocardiogram. Figure 6 shows an example of progressive fusion as a result of ventricular pacing during atrioventricular reentrant tachycardia.

## Electrocardiographic Fusion and its Potential Limitations for the Detection of Transient Entrainment

Electrocardiographic fusion is a phenomenon known for almost 100 years: a fusion complex reflects “simultaneous activation of the atria or ventricles by two, or rarely more, impulses originating in the same or, more often, in different chambers of the heart.”^9^ However, for the practical application of the concept, probably the word “impulses” could be substituted for “wavefronts” or, to be more precise, “large wavefronts.” For example, most people would agree that an example of a fused QRS complex is that observed in ventricular preexcitation, where a single impulse (a sinus beat) results in two separate (large) wavefronts that arrive in the ventricles at a similar time, so each QRS results from activation by the accessory pathway and the normal conduction system. Although fusion can also occur at the atrial level it is generally easier to observe it in the QRS complex than in the P wave. Fusion is so frequent that even the normal QRS is, to some extent, a fusion between activation due to the left and the right bundles, but since this is a normal situation, it is not usually considered under the heading of fused beats. Fused beats may occur in relation to late-coupled extrasystoles, in rhythms of ventricular origin when sinus beats capture part of the ventricles or when paced beats occur at a time when the heart has already been normally activated.

**Figure 10:**
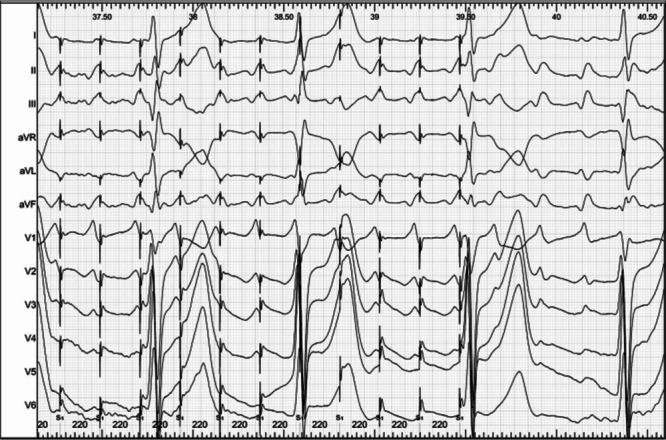
Entrainment with fusion as demonstrated by stimulation after P-wave onset. This 12-lead ECG tracing shows the last 11 beats of a pacing train at a cycle length of 220 ms delivered during an atypical flutter with a cycle length of 240 ms. The tracing is considerably gained to show P-wave morphology. Please note that each stimulus artifact is delivered after the P-wave onset, during its inscription. Capture of each stimulus can be deducted by some deformation in the P-wave contour as compared with the flutter P wave, but most importantly because the P-wave cycle length is accelerated to the pacing cycle length. Thus, since each stimulus captures but is delivered after the P-wave onset, there must be fusion, and it can be assured that fusion is due to an exiting wavefront from the tachycardia itself.

**Figure 11:**
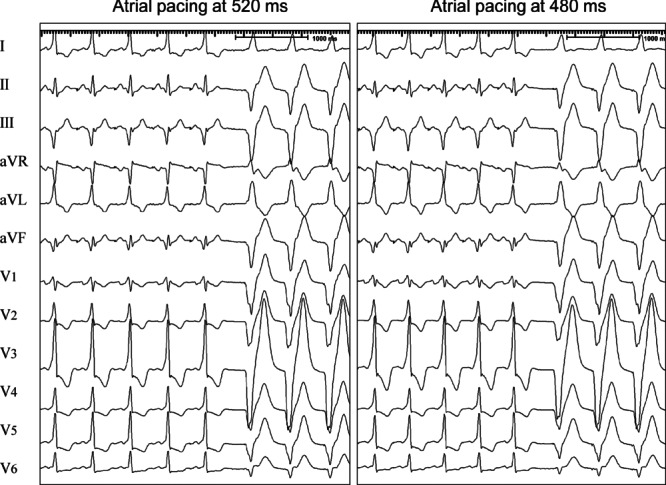
Constant and progressive fusion in the absence of entrainment and reentry. Both panels show a 12-lead ECG at the end of an atrial pacing train introduced during a “focal ventricular tachycardia” simulated by ventricular pacing in the VVI mode at a constant cycle length of 560 ms in a patient with ventricular preexcitation. In the left panel atrial pacing is performed at a cycle length of 520 ms and in the right panel at 480 ms. Note that there is constant fusion at each paced cycle length, not unexpected in preexcitation. As the pacing rate increases (right panel) there is a subtle change in the degree of fusion, as observed particularly in III, aVF, and V1. In both panels the “ventricular tachycardia” resumes after pacing is stopped. So fusion is progressive and the second criterion of entrainment met. Obviously the QRS morphology with atrial pacing at the same rate in the absence of “ventricular tachycardia” was identical, so fusion did not look at all as an intermediate morphology between tachycardia and fully paced beat. This emphasizes the importance to demonstrate that fusion is due in part to the tachycardia morphology. See text for further discussion. Courtesy of Dr. J. Garcia Fernandez.

What is specific to fusion when pacing results in resetting or entrainment, as demonstrated in Figure 8, is that the second wavefront is an exiting wavefront from the tachycardia circuit and that it collides with the paced wavefront. But, how could this be demonstrated? The only direct evidence to demonstrate this circumstance on electrocardiographic grounds (adding intracardiac recordings there are other ways; see later) is when the stimulus artifact of the paced beat occurs after the onset of the QRS or P wave (Fig. 10). The fact that the QRS/P wave onset precedes the pacing stimulus provides evidence that the tachycardia wavefront have exited from the circuit. Our group described this finding during ventricular tachycardia (VT).^6^,^10^ An indirect way to suggest this same finding would be the visual impression that the QRS- or P-wave morphology is intermediate between the tachycardia morphology and the fully paced morphology (pacing from the same site and rate in the absence of tachycardia). Finally, an even more indirect evidence would be the mere absence of other causes for fusion. Just as a remainder that pacing-related fusion not always means entrainment and reentry, Figure 11 illustrates constant and progressive fusion in the QRS developed with atrial pacing during a “focal VT” (simulated by ventricular pacing in the VVI mode in a patient with preexcitation).

Another possible mechanism of fusion unrelated to entrainment may take place if conduction in the cardiac chamber is slow enough and the pacing rate is fast enough for the activation wavefront of each beat to end after initiation of the following beat. This mechanism is more frequently detected with intracardiac recordings and will be discussed later.

When two focal rhythms at similar rate compete for the activation of a chamber (for example, a VVI pacemaker programmed at a rate similar to the sinus rate in a patient with intact conduction), there may be fused beats with beat-to-beat variation in the degree of fusion. This is usually called variable fusion and should be distinguished from constant and particularly from progressive fusion. In the latter, fusion is constant at each rate but different at different pacing rates, whereas in the former the degree of fusion varies beat-to-beat in the presence of a constant pacing rate.

Finally, since the observation of fusion implies a subjective distinction between QRS- or P-wave morphologies, it could be questioned how sensitive the electrocardiographer can be for its visual recognition. Our group has shown in a “model of ventricular fusion” that there is a correlation between the degree of fusion and its visual recognition, but that an accurate detection can only be accomplished if >20% of the QRS is fused.^11^

## Fusion looking at Intracardiac Recordings

**Figure 12:**
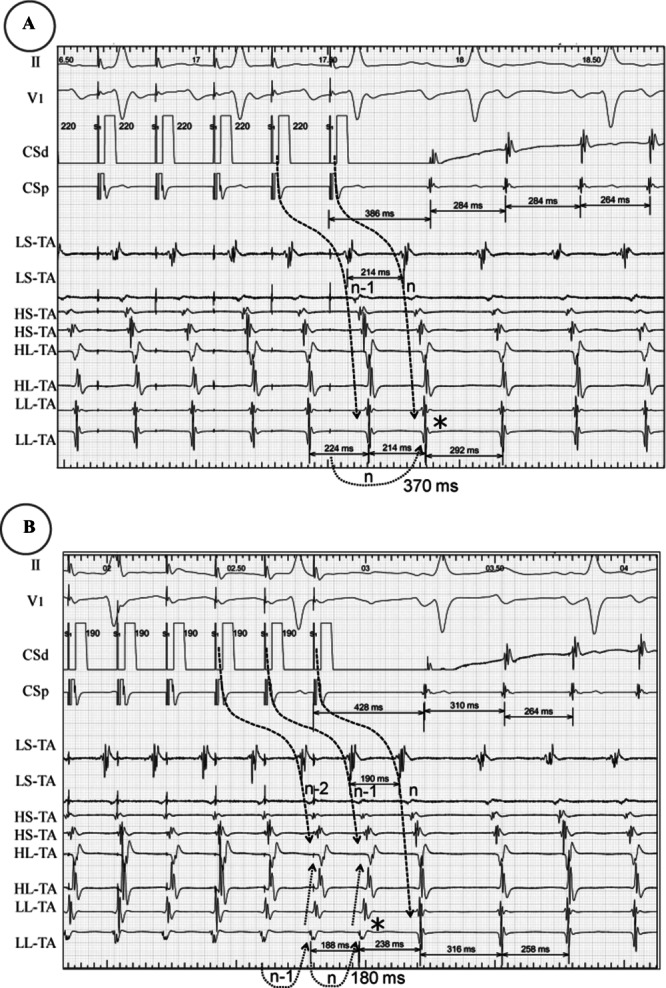
Illustrative example of the Waldo's fourth criterion for entrainment. Both panels show the end of a pacing train delivered at the distal coronary sinus (CS) during atrial tachycardia at a cycle length of 264, with resumption of tachycardia after pacing. The tracings show two surface ECG leads (II and V1) and electrogram recordings from the CS and the tricuspid annulus (TA) (from low [L] septal [S] to high [H] and low lateral). In panel A pacing is performed at 220 ms and in panel B at 190 ms. Note that counting backwards at each site allows (despite minor oscillations in conduction) assignment of each electrogram to its correspondent paced wavefront (curved descendent dashed arrow). In panel A, the TA is activated during pacing with identical activation sequence and morphology as during tachycardia (orthodromically), with a conduction time from the CS pacing site to the last TA recording (*) of 370 ms (curved ascendant arrow). In contrast, in panel B the last three TA recordings change their activation sequence and morphology during pacing (antidromic activation) as compared to tachycardia. Conduction time to the lowest AT recording (*) has now decreased to 180 ms, so it meets Waldo's fourth criterion. See text for discussion. Assignment of each electrogram to its correspondent paced beat identifies that the change in sequence is due to collision of each n antidromic wavefront to the preceding n – 1 orthodromic one.

**Figure 13:**
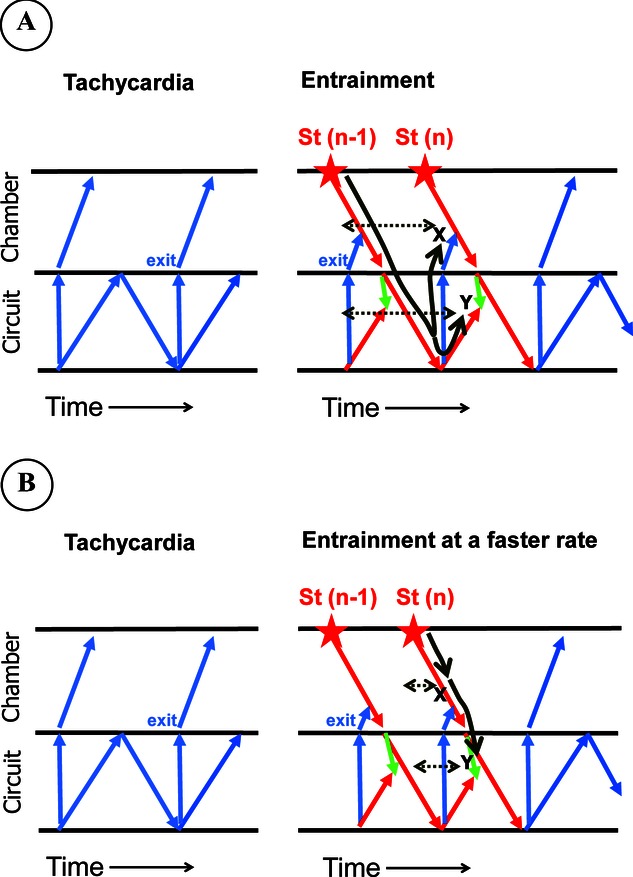
Schematic representation of the mechanism of Waldo's fourth entrainment criterion. The format is similar to that of Figure 7. Sites X and Y represent sites inside and outside the circuit that are activated orthodromically at the slower rate (panel A) but antidromically at a faster rate (panel B). The black solid manual line represents the route of activation from the paced site to the recorded site and the dashed gray arrows the conduction time from the stimulus to the recorded site. Comparing panels A and B it can be noted that a faster pacing rate is translated into a dramatically shorter conduction time to both sites X and Y.

From the above discussion, it is clear that fusion may be sometimes difficult to recognize in the surface electrocardiogram, so it would be useful if the intracardiac recordings can help. Henthorn et al. described what they called a fourth criterion for the recognition of transient entrainment (they had described a third criterion in relation to tachycardia termination; see part II of this review): “during a tachycardia, when pacing at two constant rates that are faster than the rate of tachycardia, but which fail to interrupt it, there is the demonstration of a change in conduction time to and electrogram morphology at an electrode recording site.”^12^ And they added: “this is the electrogram equivalent of progressive fusion.” Figure 12 shows an example of this criterion. Since conduction velocity with increasing rate is expected to either stay the same or decrease, but not increase, a decrease in conduction time (same paced site, same recorded site) in relation to faster pacing rate demonstrates that there are two routes of activation and that the faster one can only conduct to the recording site at faster pacing rates. The dynamics of entrainment offer a good explanation for that finding: the amount of tissue antidromically captured (both inside and outside the circuit) is critically dependent on the pacing rate, as shown in Figures 5 and 9. If the recording site is located in an area activated orthodromically at a slower rate and antidromically at a faster rate, the conduction time will dramatically shorten at the faster pacing rate (Fig. 13). Adequate measurement of intervals is critical for the application of this criterion. The only way to deduct which paced wavefront activates a particular site, as shown in Figure 12, is to measure intervals between electrograms immediately after pacing to find out when the local cycle length differs from the paced cycle length (PCL).

The above discussion provides clues to the strength, but also to the limitations, of this criterion; as shown in Figures 5 and 9 the amount of tissue (both inside and outside the circuit) where this phenomenon occurs is really limited, so the recording site has to be located “at the right place.” The way to maximize this possibility is to increase the difference in pacing rate between the slower and the faster pacing rate, but in clinical electrophysiology this is limited because of tachycardia termination or degeneration into a different arrhythmia by this maneuver.

**Figure 14:**
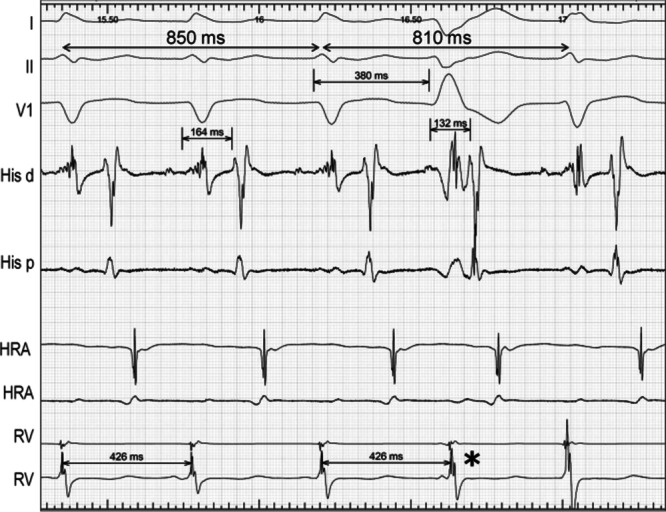
Resetting with fusion as demonstrated by the local electrogram. The tracing shows three surface ECG leads and intracardiac recordings from His, high right atrium (HRA), and right ventricle (RV) during a regular accessory pathway-mediated supraventricular tachycardia. The fourth QRS complex is a left ventricular spontaneous extrasystole that results in resetting of the tachycardia since the interval between the two QRS encompassing the extrasystole (810 ms) is shorter than two tachycardia cycle lengths (850 ms). The fact that the RV electrogram (*) is not changed in timing or morphology demonstrates that there is ventricular fusion.

**Figure 15:**
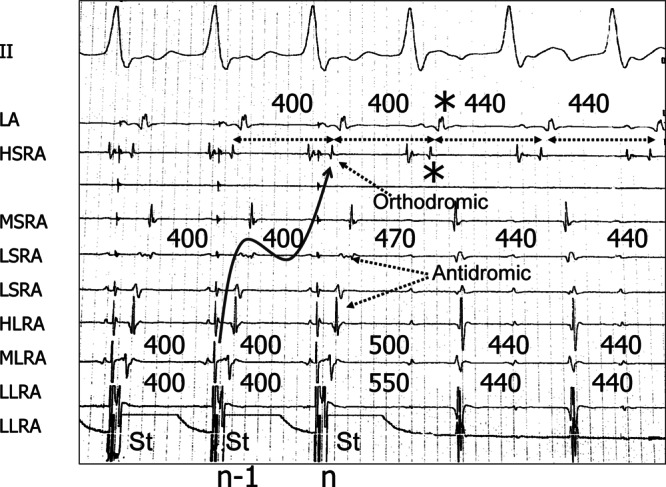
Entrainment of a reentrant atrial tachycardia demonstrating fusion by timing (and morphology) of local electrograms. The tracing shows surface ECG lead II and intracardiac recordings from the left atrium (LA) and the high (H), mid (M), and low (L) septal (S) and lateral (L) right atrium (RA) during the last three beats of a pacing train delivered at the LLRA during tachycardia, that continues after pacing is stopped. Measuring consecutive intervals at the HSRA and LA shows that they are activated at the pacing cycle length up to the marked beat (*). So this is the result of the n-paced beat. The previous electrogram at HSRA and LA is the result of the n – 1 beat (as depicted by the curvilinear arrow) but occurs after the stimulus of the n beat demonstrating the temporal coexistence of two activating wavefronts, that is, fusion. Furthermore, in this particular example HSRA has two components that encompass the n stimulus, having identical morphology and timing during tachycardia (orthodromic). Please note that intervals at orthodromic sites go from PCL to TCL without transitional interval. In contrast, intervals at antidromically activated sites (LLRA, MLRA, HLRA, LSRA) go from paced cycle length (400 ms) to tachycardia cycle length (440 ms) having a transitional interval in between, that is different (in this case longer) from both. See text for discussion.

**Figure 16:**
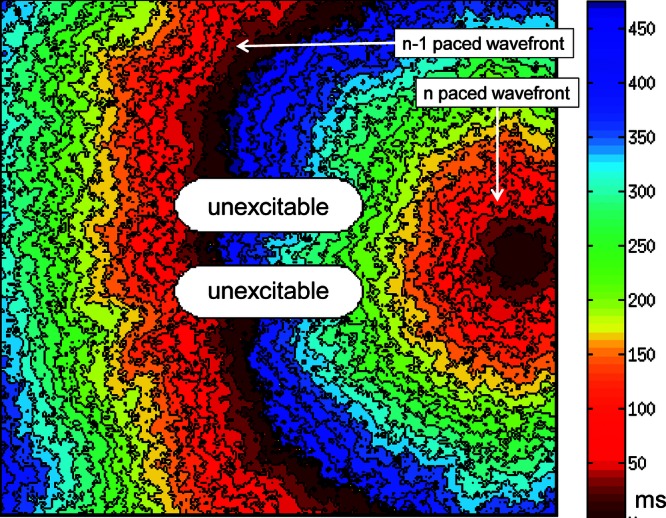
Fusion without collision. The computer simulation illustrates the result of simple pacing in the setting of extremely slow conduction. Note that two wavefronts coexist at the same time because the previous wavefront has not finished the activation of the chamber when a new wavefront appears.

**Figure 17:**
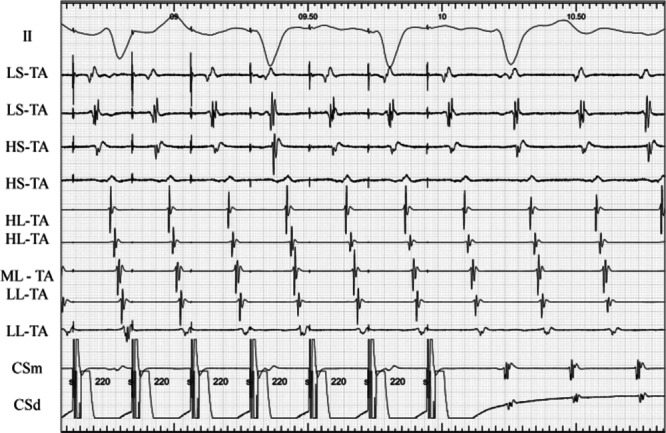
Unexpected sequence according to anatomy suggests fusion and entrainment. The tracing shows surface ECG lead II along with intracardiac bipolar recordings from the tricuspid annulus (TA) (from low [L] septal [S] to high [H] and low lateral) and the mid (m) and distal (d) coronary sinus (CS). The last several beats of a pacing train at a cycle length of 220 ms, delivered during atrial flutter, are shown with continuation of flutter. Note that, despite an acceleration of the recordings of the TA to the pacing rate, the activation sequence of the low and mid lateral TA is unexpected for a CS pacing location. This mere finding is suspicious of constant fusion and thus, entrainment. In this particular case, the identity between the activation sequence during pacing and during tachycardia accentuates this suspicion.

**Figure 18:**
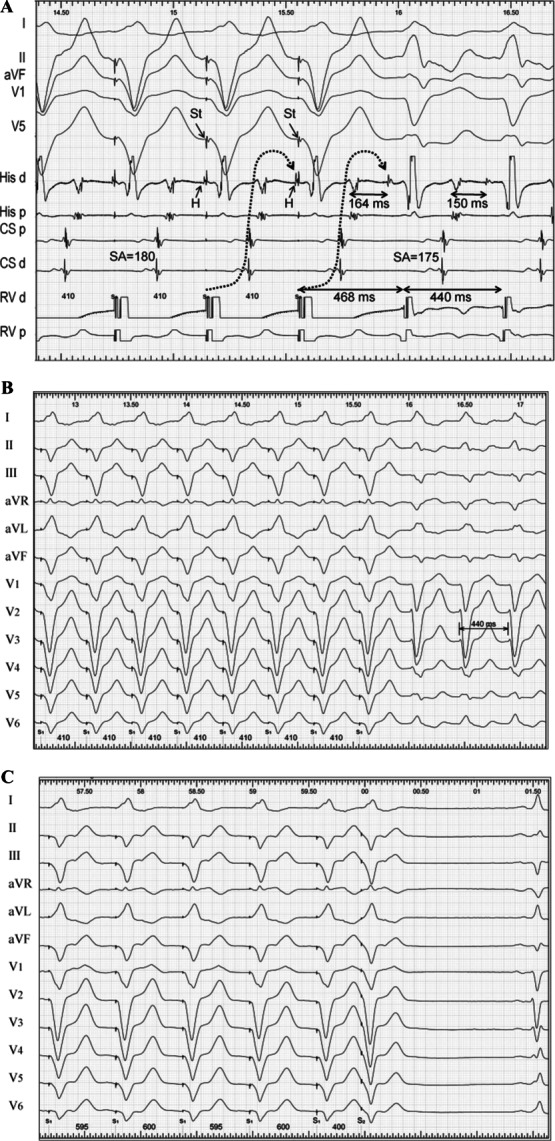
Entrainment without fusion. Panels A and B shows the last several beats of a right ventricular pacing train at a cycle length of 410 ms, delivered during an orthodromic left free-wall accessory pathway-mediated tachycardia with left bundle branch block at a cycle length of 440 ms. Panel C shows right ventricular pacing at a cycle length of 600 ms followed by an extrastimulus at 400 ms delivered at the same site as in panels A and B but during sinus rhythm. Panel A shows five surface ECG leads and intracardiac recordings from distal (d) and proximal (p) His, coronary sinus (CS), and right ventricular apex (RV). Panels B and C show 12-lead ECG. Panel A demonstrates acceleration to the pacing rate with orthodromic capture of all atrial electrograms and the His bundle (curved arrow). The postpacing interval exceeds the tachycardia cycle length only by 28 ms, and the postpacing atrial-His (AH) exceeds the tachycardia AH by 14 ms. The stimulus-atrial and ventriculoatrial intervals are also virtually identical. The QRS morphology during pacing and entrainment of the tachycardia in panel B is identical in the 12-lead ECG to that of pacing in the absence of tachycardia as shown in panel C.

Intracardiac recordings can help in other ways as well:

1. Resetting of tachycardia in the presence of local electrograms without a change in timing and morphology. In Figure 14 resetting demonstrates the interaction between the extrasystole and the basic rhythm, and fusion is demonstrated because some areas of the ventricles (at least the apex of the RV where the local electrogram is located) occur at the tachycardia rate and with identical morphology (the tachycardia is still alive).
2. Orthodromic activation of areas in relation to the n – 1 paced beat coincidental in time with areas that are activated by the n paced beat (Fig. 15); this is in itself a demonstration of fusion since two wavefronts (n – 1 and n) activate the same chamber at the same time; it is an important phenomenon because it can be frequently observed. The only caveat to this phenomenon is that it can also be observed if conduction is so slow (and/or pacing or the intrinsic rhythm so fast) that the activation of the chamber occurs as a result of two different unrelated wavefronts. The computer simulation presented in Figure 16 represents a focal mechanism in the presence of an extremely slow conduction (the model is the same as that in Figure 4B but conduction velocity is markedly slowed). This promotes that each beat starts while the preceding wavefront is still activating the chamber, so two wavefronts coexist temporally, that is, there is fusion. One can imagine that if a paced rhythm interacts with (depolarizes) the focus, fusion will still be observed (and it is not a reentrant rhythm). However, in this situation, there is no collision between n – 1 and n wavefronts and fusion is a mere “bystander phenomenon” (bystander fusion). This emphasizes how important is to demonstrate collision, which may not be an easy task. Useful proposals to avoid the spurious fusion presented in Figure 16 include: (a) identification of fusion close to the reentrant circuit, as opposed to far from the origin of impulses, as seen in Figure 16; (b) avoid pacing much faster than the tachycardia rate, as this could produce marked delays in conduction as has been shown in common flutter^13^; (c) be particularly careful when mapping during the original rhythm already shows fusion (activation at the same time of sites located far away).
3. Unexpected sequence according to anatomy. Figure 17 illustrates this phenomenon. In normal conditions and during sinus rhythm, when pacing from the coronary sinus, the infero lateral tricuspid annulus activates in a caudocranial sequence, just because its inferior aspect is anatomically closer to the coronary sinus. However, in Figure 17 coronary sinus pacing results in a descending lateral tricuspid annulus activation sequence. This should make us suspect that another wavefront is going on, preventing tricuspid annular activation as expected from coronary sinus pacing. If that were the case, this would be a demonstration of fusion. However, this criterion per se, has to be used with great caution: one has to be sure that catheter locations are stable and that conduction velocity is not regionally impaired; otherwise, this criterion could be misleading.
4. Acceleration of the whole chamber in which reentry is located, including the tissue in the reentrant circuit, to the pacing rate, without tachycardia termination. Our group has used this criterion in the setting of accessory pathway-mediated tachycardia and atrioventricular (AV) nodal reentrant tachycardia.^14^ The reasoning behind this use is that, if the acceleration of a critical part of the circuit is guaranteed and the tachycardia is not terminated, the chances of not having been entrained are very little; only termination and reinitiation can do this, and this possibility is minimized if the maneuver is repeated. Figure 18A illustrates this finding in an accessory pathway-mediated tachycardia; continuous ventricular pacing results in acceleration of ventricles and atria to the pacing rate. This guarantees continuous 1:1 interaction between all cardiac chambers and critical components of the reentrant pathway. Tachycardia persistence is shown by its continuation when pacing is stopped. In this particular instance, even the His bundle recording is orthodromically activated, demonstrating also that the AV node has been accelerated to the pacing rate. This guarantees that continuous 1:1 interaction between all cardiac chambers and all components of the reentrant pathway has taken place. This fulfills the essence of all the entrainment phenomena. In relation to resetting the possibility of termination and reinitiation is out of consideration since only one beat interacts with the tachycardia, so the finding is even more firm as evidence of interaction, but since only one beat interacts with the circuit, it will not be activated at the pacing rate; in this situation, an advancement of the activation will represent the same finding.

## Resetting and Entrainment without Fusion: Concealed Entrainment

**Figure 19:**
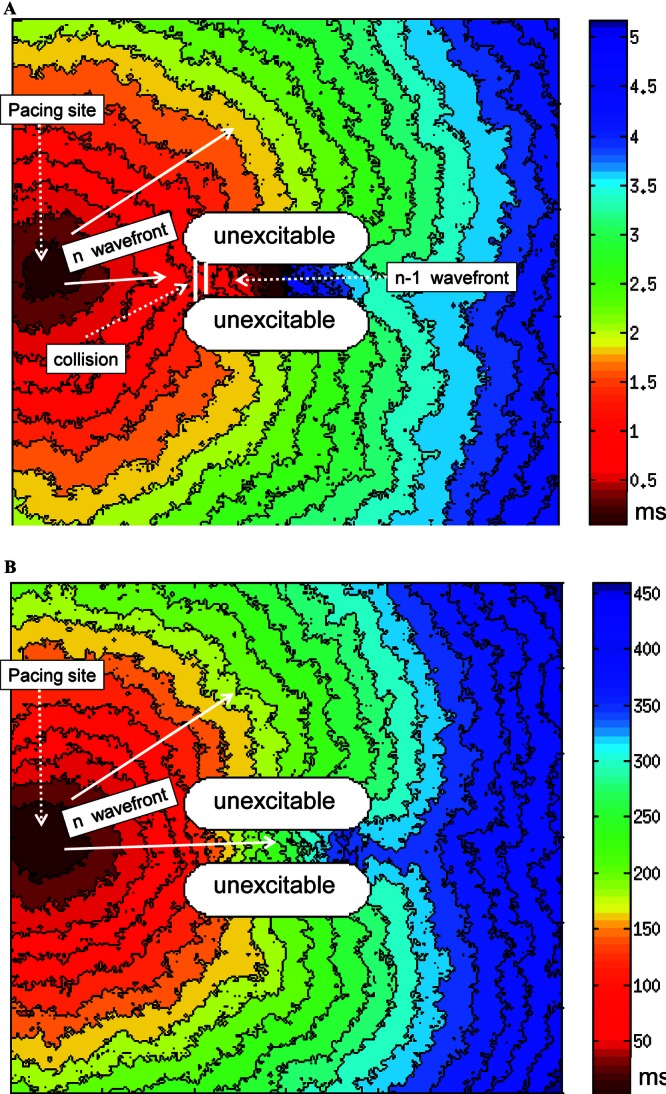
Computer simulation of entrainment without fusion. Panel A: activation during a paced beat entraining the reentrant rhythm, as seen in Video 2. The format is similar to that in Figure 4A. Panel B: activation of the same preparation by a paced wavefront generated at the same site as in panel A, but in the absence of reentry, as seen in Video 3. Please note that activation is identical except for the tissue in between the two anatomical barriers (the protected area), that during entrainment is activated from right to left with collision between the n – 1 wavefront and the n wavefront, and this is not the case in panel B.

Figure 18B shows the 12-lead ECG of the entrainment tracing of Figure 18A. The QRS morphology in all 12-lead ECGs during entrainment is identical to that of pacing in the absence of tachycardia as shown in Figure 18C. How can we be sure that the tachycardia has been entrained if none of the four described criteria are met? In this particular instance, as discussed above, because we can record from “inside” the circuit and demonstrate the essence of entrainment. Thus, entrainment can occur despite the absence of the reported criteria. The mechanism of this type of entrainment is illustrated in the computer simulation (Videos 2 and 3, Fig. 19). It can be appreciated that there is, in fact, fusion and collision (Fig. 19A); however, it is restricted to part of the protected area, so it is unlikely that it will change the configuration of the surface ECG (this could be considered concealed fusion, although this is not what is usually considered as such; see later). Thus, the ECG morphology during entrainment will be identical to that of pacing in the absence of tachycardia (Fig. 19B).

**Figure 20:**
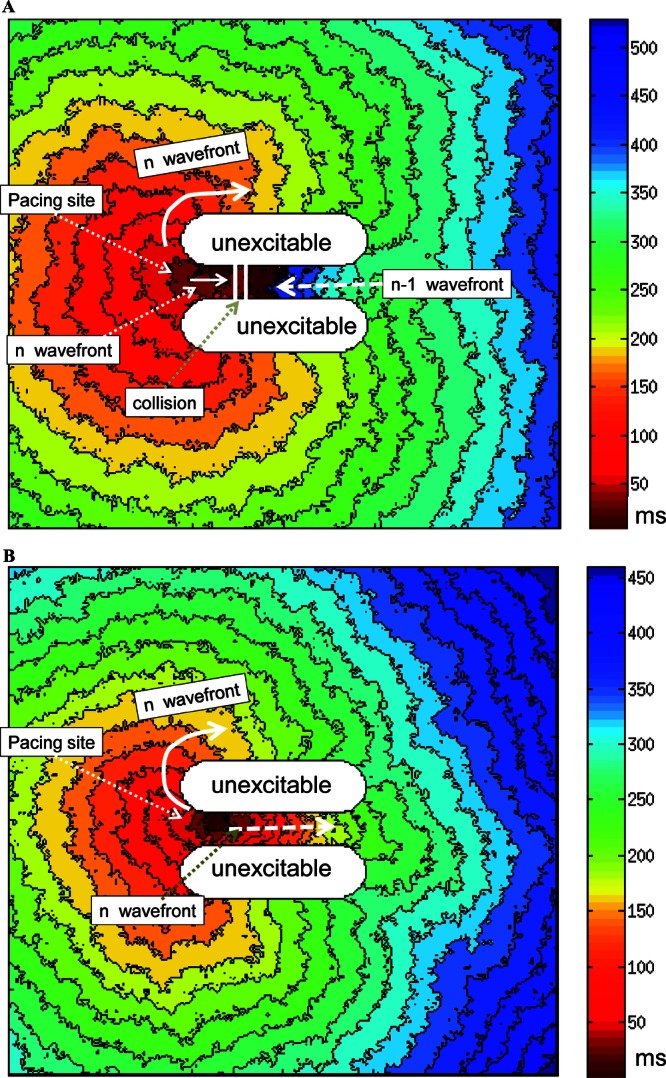
Computer simulation of concealed entrainment. Panel A: activation during a paced beat entraining the reentrant rhythm, as seen in Video 4. The format is similar to that of Figure 4A, but pacing is performed within the protected area. Panel B: activation of the same preparation by a paced wavefront generated at the same site as in panel A, but in the absence of reentry. Please note that in panel A activation is identical as in tachycardia (Fig. 4A) except for the tissue in between the two anatomical barriers (the protected area), that during entrainment is activated partially from right to left with collision between the n – 1 wavefront and the n wavefront. In contrast to Figure 19, now during pacing in the absence of tachycardia (panel B), some tissue at the right of the protected area (and outside of it) is activated differently.

**Figure 21:**
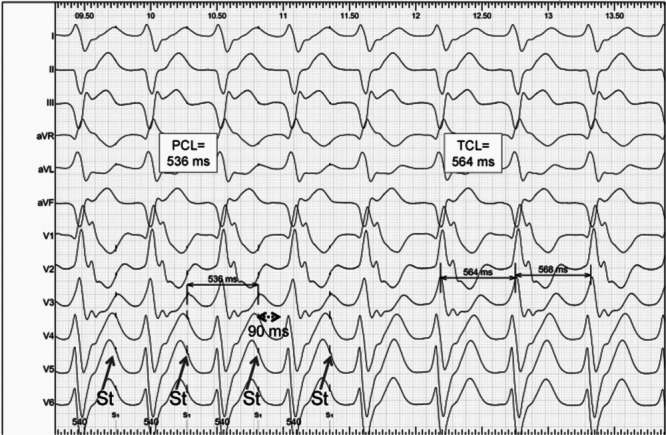
Example of concealed entrainment during ventricular tachycardia. This 12-lead ECG shows the last four beats of left ventricular pacing train at a paced cycle length (PCL) of 536 ms delivered during a slow ventricular tachycardia with a tachycardia cycle length (TCL) of 564 ms. Please note that sustained capture is demonstrated because the QRS are consistently activated at the PCL with identical stimulus (St) to QRS onset. Also note that stimulus to QRS onset is long (90 ms). But despite capture, the paced QRS morphology is identical in all 12 leads to the tachycardia QRS morphology. This tracing, by strict criteria, does not prove entrainment, because there is no proof of interaction between the paced wavefronts and the tachycardia, and, obviously none of the entrainment criteria are met.

A somewhat different mechanism could also lead to absence of electrocardiographic fusion, but in a different way, during resetting and entrainment. If pacing is performed during tachycardia from the protected area (see Video 4, Fig. 20A), collision will only take place inside the protected area and the paced wavefront is “forced” to exit and activate the chamber in an identical manner as during tachycardia. This is a unique situation because the ECG morphology during entrainment is indistinguishable from that during tachycardia (Fig. 21). In addition, since the paced wavefront arises from the protected area, that lacks electrocardiographic representation, there is usually a considerable time elapsed between the stimulus and its electrocardiographic consequence, the paced wavefront. This association between an identical ECG morphology during entrainment and tachycardia and a long stimulus to QRS- or P-wave onset has been called “concealed entrainment” or, more appropriately, “entrainment with concealed fusion.” Interestingly enough, however, pacing from the same site in the absence of tachycardia could produce a different morphology (Fig. 20B) as long as antidromic conduction through the protected area can occur. It should be noted, and will be discussed in the second part of this review, that the same phenomenon of concealed entrainment will occur if pacing is performed at a site that is not part of the reentrant circuit, but is protected and connected to a protected area of the circuit (“bystander site”).

These two scenarios illustrate two different ways in which entrainment without fusion can occur. In both situations fusion does occur but it is restricted to the protected area so it lacks electrocardiographic representation. In the former situation fusion is lacking because the entrained chamber is activated in an identical manner as the paced chamber in the absence of tachycardia (fully paced QRS or P wave); in the latter situation fusion is lacking because the entrained chamber activates in an identical manner as during tachycardia. Despite the similarities between both situations (fusion is concealed in both), only the latter has been traditionally considered as “concealed entrainment,” because it was originally studied in the context of VT origin localization (see the second part of this review). It should be realized that in both situations it would be difficult to demonstrate any of the described entrainment criteria. Then, how could we know that entrainment occurred? In some cases, as discussed before, recordings from inside the protected area could provide evidence that the “essence” of the phenomenon has occurred. Otherwise, clues may be obtained by some additional criteria, as suggested in the second part of this review.

## Determinants of Fusion during Resetting and Entrainment

From the above discussion it is clear that resetting and entrainment can occur both in the presence and in the absence of electrocardiographic fusion. Since fusion facilitates the recognition of entrainment, it is pertinent to ask for the determinants of its presence. The comparison of Figures 5A and 9A illustrates that entrainment with and without fusion can occur in the same chamber and circuit but with different location of the pacing sites. Based on the observation of the absence of ventricular fusion during ventricular entrainment of accessory pathway-mediated tachycardia (and its presence during atrial pacing), Okumura et al. proposed that presence or absence of fusion during entrainment was related to pacing location proximal or distal to the area of slow conduction (the AV node in this case).^15^ We have shown, however, that other factors can influence this phenomenon; in the same “model” of accessory pathway-mediated tachycardia, we showed that right ventricular stimulation can entrain with electrocardiographic fusion as long as the pathway is located in the septum or in the right free wall.^14^ Figure 6 is an example. This demonstrates that factors other than the location proximal or distal to the area of slow conduction influence overt fusion. In fact, even in left free wall orthodromic accessory pathway-mediated tachycardia, ventricular pacing (so distal to the area of slow conduction) can demonstrate entrainment with fusion if pacing is performed from the left ventricle (Fig. 7). Our group showed that fusion during entrainment of VT was related to the antidromic capture of presystolic electrograms; fusion was always absent if presystolic electrograms were antidromically captured.^8^ The scheme shown in panels B and C of Figure 8 illustrate why this happens: if the exit site is antidromically invaded before the activation wavefront has exited from the protected area, no fusion could be observed on the ECG. We proposed that the relative localization of pacing site relative to entrance and exit from the (protected area) circuit determines electrocardiographic fusion. This is also illustrated by the influence of the type of circuit, as regard to entrance and exit site, in relation to resetting and entrainment with fusion. For example, the exit of AV nodal reentrant tachycardia on its distal end is the His bundle (with or without a lower final common pathway). Pacing from the ventricle, in the absence of accessory pathways, the entrance to the AV nodal reentrant tachycardia circuit is also the His bundle. Since entrance and exit are the same, ventricular fusion was predicted to be absent, and was in fact found to be absent.^8^ Another interesting situation in this respect is when the circuit “enlarges” but the geometric disposition of pacing site and entrance to and exit from the circuit remain unchanged. A frequent example of this kind occurs in left free wall orthodromic accessory pathway-mediated tachycardias when left bundle brunch block occurs and pacing is performed from the right ventricular apex. Since the circuit is now closer to the pacing site, resetting becomes “easier” and occurs with late-coupled extrastimuli, but fusion does not occur because the exit site (the right bundle in this case) is so close to the pacing site that is likely to be antidromically invaded. Figure 1 (bottom panel) shows an illustrative example; note that the tachycardia is reset with a late-coupled extrastimulus but the morphology of the paced QRS does not show fusion; it looks like “fully paced apical pacing” (although stimulation during sinus rhythm is not shown for comparison). Finally, dynamic determinants of fusion are pacing rate (relative to tachycardia rate) and decremental conduction inside the circuit. The ladder diagrams of Figures 8A and B clarify that less fusion will occur as the pacing rate increases, to the point of absence of fusion at a certain rate. Figure 6 illustrates that pacing at a faster rate (right panel) approximates the expected paced QRS from the right ventricular apex so there is less fusion. As discussed above, more antidromic invasion implies less or absent fusion. If conduction of the orthodromic wavefront slows down in relation to pacing rate (decremental conduction) it will arrive later to its destination to collide with the antidromic wavefront, resulting in more antidromic invasion and less and eventually no fusion.

## Differences between Resetting and Entrainment

As discussed above, the two phenomena are essentially similar in their mechanisms and implications. However, they differ in several theoretical and practical aspects.

The resetting phenomenon can easily be recognized by comparing the intervals encompassing the extrastimuli with the TCL (Fig. 1). If they differ from twice the TCL (in the case of a single extrastimulus) or from three times the TCL (in the case of double extrastimuli) the tachycardia has been reset. As discussed above, the situation is different for the entrainment response, we cannot use intervals, and thus other criteria are needed, more difficult to observe and demonstrate.

Resetting may be more limited than entrainment when there is a long conduction time between the stimulation site and the circuit. Since there are wavefronts exiting from the tachycardia, one or even two extrastimuli may collide with such wavefronts outside the circuit, preventing an interaction with it. Our group studied this phenomenon during stable sustained VT initiated by programmed electrical stimulation.^10^ It was found that resetting occurred in 55% and 79% of tachycardias in response to single and double right ventricular extrastimuli, respectively. We proposed that a single ventricular extrastimulus that did not reset the tachycardia could produce two important changes: (1) reversal of the activation sequence in part of the intervening myocardium between the tachycardia origin and the pacing site; (2) shortening of the local effective refractory period at the pacing site. Both of these changes facilitated the second extrastimulus accessing and interacting with the circuit, explaining the higher incidence of resetting with double extrastimuli. As pacing is continued searching for the entrainment response, conduction time between the pacing site and the circuit will no longer be a limiting factor for accessing the circuit and eliciting the entrainment response.

The resetting response induces less modification in the dynamics of circuit.^16^ And this is so for two reasons: (1) it interacts with the circuit only once, so the interaction takes place with the “original reentrant wavefront.” In contrast, during entrainment there is repetitive resetting, so the interaction of each wavefront occurs with the previously reset circuit. (2) It interacts with the circuit with the lowest possible prematurity; using a strict protocol (see later), the latest coupled extrastimulus that resets the circuit will enter the circuit with the minimum possible degree of prematurity, and so will advance the activation as little as possible, inducing the least possible modification in the reentrant pathway. In contrast, by continuous pacing at a rate somehow faster than the tachycardia rate, the degree of prematurity of the first beat that interacts (and resets) with the circuit will be unknown (a value in between the difference between the tachycardia and the PCL).

Pacing maneuvers can modify the original arrhythmia and induce other undesired arrhythmias. It is hard to know if this risk is higher with extrastimuli or with continuous pacing. On the one hand for extrastimuli to produce resetting short coupling intervals may have to be reached. On the other hand, continuous pacing, although with a longer cycle length, will interact more with the circuit that could be destabilized.

A more practical issue can also influence the choice of pacing maneuver: extrastimuli have to be appropriately synchronized in order to produce resetting. When pacing is attempted from a low voltage area, synchronization may not be accurate, since some commercial stimulators can only sense at the same site where they pace and the highest sensitivity value may be as low as 1 mV. In such circumstances, some electrophysiologists may choose asynchronous continuous pacing that can always produce entrainment. However, it has to be realized that in the latter case, the coupling interval of the first stimulus interacting with the circuit is totally uncontrolled.

## Pacing Protocol for Resetting and Entrainment

Protocol for studying resetting: we first introduce single extrastimuli during tachycardia, starting at a coupling interval 10 ms less than the TCL, and decreasing the coupling interval of the subsequent extrastimuli by 10 ms steps until resetting occurs or local refractoriness is reached. At least eight spontaneous beats are allowed in between extrastimuli to avoid modifications in the tachycardia. If resetting does not occur with one extrastimulus, double extrastimuli are programmed, with the coupling interval of the first constant and 10–20 ms above refractoriness. If resetting does occur with one extrastimulus, double extrastimuli are programmed, with the coupling interval of the first constant and 10 ms longer than the longest coupling interval that produced resetting with single extrastimuli. The coupling interval of the second extrastimulus will be started equaling the TCL, and decreased in 10 ms decrements until resetting or refractoriness. For interpretation, always check for morphologic variations in the first or second beat after pacing. They are relatively frequent and in such cases the maneuver has to be repeated.

Protocol for studying entrainment: in order to avoid altering the circuit, pacing should be performed in a synchronous fashion and at a PCL close to the TCL. We start with a PCL 20–30 ms less than the TCL; less than that makes it difficult to recognize capture and entrainment. The number of paced beats is usually started at 10 and increased if necessary. A particular protocol for the recognition of entrainment involves delivering different pacing trains increasing the number of beats on each train (it will be presented in the second part of this review).

The pacing output for both resetting and entrainment will ideally be well over threshold (to ensure continuous capture) but not too high to avoid a large virtual electrode. In addition, if threshold has not been measured in the absence of tachycardia, threshold measurements during tachycardia may produce asynchronous pacing (or undesired entrainment), which could alter the tachycardia. Depending upon the case, a reasonable compromise to approximate threshold measurements in a rapid fashion could be to deliver late coupled extrastimuli at a constant coupling interval, starting at 3–5 mA and increasing by 2–3 mA steps.

## Supporting Information

The following supporting information available for this article:

**Video 1**. Computer simulation of a two-dimensional “figure-of-eight” reentrant arrhythmia, during which stimulation away from the circuit results in entrainment with manifest fusion. After initiation of reentry and two reentrant beats, pacing is initiated at the right margin of the planar model. The first paced beat does not reset the tachycardia, but the second beat does, and the third, fourth, and fifth beats entrain it. Note that there is manifest fusion in that a big area surrounding the pacing site is activated by the paced wavefront, but the rest of the model keeps activating as during tachycardia. Collision between these two activation wavefronts can be easily appreciated for each paced beat. For this and the subsequent videos, the Automata model was built with a rectangular bidimensional substrate representing a cardiac tissue planar section, subsequently subdivided in small cell groups. The reaction process of each cell is described by means of three different states and three transitions, according to a rest, activated, and refractory characteristics. The transitions in response to reaction were modeled using restitution curves of the action potential duration vs the diastolic interval and the conduction velocity. This model has been previously shown to be suitable for considering reentry, fibrillatory, and other arrhythmic mechanisms.^17^ The diffusion process was modeled with a mixed linear (impulse response of charge) and nonlinear (threshold effect) jointly considered at each integration step, which represents a significant improvement over our previous version.

**Video 2**. Computer simulation of entrainment without fusion. The format and reentrant arrhythmia are identical to that in Video 1. Likewise, reentry is initiated and, after two reentrant beats, pacing is initiated at a constant rate. In contrast to Video 1, the pacing site is now at the left margin of the planar model. In this case, the first paced beat resets the tachycardia, and the following entrain it. Note that, except for the small protected area located inside the two anatomical barriers, the remaining of the planar model activates during pacing in identical manner as expected if the tachycardia was absent (“fully paced” activation; see Video 3), so there is no overt fusion.

**Video 3.** Computer simulation of pacing from identical site as in Video 2, but in the absence of reentry. The format of the computer simulation is identical to that in Video 1.

**Video 4.** Computer simulation of concealed entrainment. The format and reentrant arrhythmia are identical to that in Video 1. Likewise, reentry is initiated and, after two reentrant beats, pacing is initiated at a constant rate. In contrast to Videos 1 and 2, the pacing site is now at the area located inside the two anatomical barriers. In this case, the first paced beat resets the tachycardia, and the following entrain it. Note that, except for a small part of the protected area inside the two anatomical barriers, the planar model activate during pacing in identical manner as during tachycardia, so there is no overt fusion.
